# Osimertinib as Second- and ≥Third-Line Treatment in Advanced and Recurrence EGFR-Mutant NSCLC Patients Harboring Acquired T790M Mutation

**DOI:** 10.3390/cancers16244174

**Published:** 2024-12-14

**Authors:** Mu-Han Peng, Yen-Hsiang Huang, Kuo-Hsuan Hsu, Jeng-Sen Tseng, Po-Hsin Lee, Kun-Chieh Chen, Gee-Chen Chang, Tsung-Ying Yang

**Affiliations:** 1 Department of Chest Medicine, Taichung Veterans General Hospital, No. 1650, Sect. 4, Taiwan Boulevard, Taichung 407, Taiwan; fox0714@vghtc.gov.tw (M.-H.P.); khhsu@vghtc.gov.tw (K.-H.H.); tzeng64@vghtc.gov.tw (J.-S.T.); berry7bo@yahoo.com.tw (P.-H.L.); tyyang@vghtc.gov.tw (T.-Y.Y.); 2 Lung Cancer Comprehensive Care and Research Center, Taichung Veterans General Hospital, No. 1650, Sect. 4, Taiwan Boulevard, Taichung 407, Taiwan; 3 Faculty of Medicine, School of Medicine, National Yang Ming Chiao Tung University, No. 155, Sect. 2, Linong St., Taipei 112, Taiwan; cshy1888@csh.org.tw; 4 Department of Post-Baccalaureate Medicine, College of Medicine, National Chung Hsing University, No. 145, Xingda Rd., South Dist., Taichung 402, Taiwan; 5 Institute of Biomedical Sciences, National Chung Hsing University, No. 145, Xingda Rd., South Dist., Taichung 402, Taiwan; 6 Doctoral Program in Translational Medicine, National Chung Hsing University, No. 145, Xingda Rd., South Dist., Taichung 402, Taiwan; 7 Rong Hsing Translational Medicine Research Center, National Chung Hsing University, No. 145, Xingda Rd., South Dist., Taichung 402, Taiwan; 8 Division of Pulmonary Medicine, Department of Internal Medicine, Chung Shan Medical University Hospital, No. 110, Sec. 1, Jianguo N. Road, Taichung 402, Taiwan; ckjohn@mail2000.com.tw; 9 Institute of Medicine, Chung Shan Medical University, No. 110, Sec. 1, Jianguo N. Road, Taichung 402, Taiwan; 10 School of Medicine, Chung Shan Medical University, No. 110, Sec. 1, Jianguo N. Road, Taichung 402, Taiwan

**Keywords:** epidermal growth factor receptor, tyrosine kinase inhibitor, osimertinib, non-small-cell lung cancer, T790M

## Abstract

Background/Objectives: Osimertinib is a standard sequential therapy for advanced and recurrent Epidermal Growth Factor Receptor (EGFR)-mutant non-small-cell lung cancer (NSCLC) patients with the T790M mutation, following treatment with first- or second-generation EGFR Tyrosine Kinase Inhibitors (TKIs). This study aims to investigate the differences in clinical outcomes between osimertinib as a 2nd-line treatment and as a ≥3rd-line treatment in this patient population. Methods: Between September 2014 and March 2023, we enrolled advanced and recurrent T790M + NSCLC patients who had received osimertinib as sequential treatment for analysis. All patients had previously been treated with gefitinib, erlotinib, or afatinib as first-line therapy. Results: A total of 158 patients who received osimertinib as sequential treatment were included in the final analysis. Of these, 99 patients (62.7%) received osimertinib as a 2nd-line treatment, while 59 patients (37.3%) were treated with osimertinib as ≥3rd-line therapy. The median progression-free survival (PFS) was 10.7 months for the 2nd-line group and 8.9 months for the ≥3rd-line group. The median overall survival (OS) from first-line treatment was 73.2 months in the 2nd-line group and 57.5 months in the ≥3rd-line group. No statistically significant differences in PFS or OS were observed between the two groups. Conclusions: Our research demonstrated that osimertinib is effective not only as a 2nd-line therapy but also as a ≥3rd-line treatment, offering promising clinical benefits for advanced and recurrent EGFR-mutant NSCLC patients with acquired T790M mutations who have developed resistance to first- and second-generation EGFR-TKI therapy.

## Simple Summary

The objective of this study was to compare the clinical outcomes of osimertinib as a 2nd-line treatment versus as a ≥3rd-line treatment in advanced and recurrent Epidermal Growth Factor Receptor (EGFR)-mutant non-small-cell lung cancer (NSCLC) patients with acquired T790M mutations. A total of 158 patients who received osimertinib as sequential treatment were enrolled for final analysis between September 2014 and March 2023. Among these, 99 patients (62.7%) received osimertinib as 2nd-line treatment, while 59 patients (37.3%) were treated with it as ≥3rd-line therapy. We found no significant difference in progression-free survival or overall survival between the two groups. Our findings suggest that osimertinib is not only effective as a 2nd-line therapy but also as a ≥3rd-line treatment, offering promising clinical benefits for advanced and recurrent EGFR-mutant NSCLC patients with acquired T790M mutations.

## 1. Introduction

Lung cancer is one of the leading causes of mortality in the world. Among the overall population, non-small-cell lung cancer (NSCLC) accounts for the majority of patients diagnosed with lung cancer, according to the histologic types. Currently, the treatment of lung cancer lies in the era of personalized and precision medicine. The therapeutic strategy for lung cancer is based not only on the results of histology but also on molecular biology tests. According to a previous study, advanced NSCLC patients harboring druggable oncogenic driver mutations experienced better overall survival (OS) than those without driver mutations if they had previously received genotype-directed therapy [1]. Thus, driver gene-based target therapy has become the cornerstone for lung cancer treatment.

The distribution of oncogenic driver mutation is very different between races. For Caucasians, the majority in driver mutation type is the KRAS mutation, accounting for approximately 25% of NSCLC patients [2]. On the other hand, there is an approximately 50–60% rate of Epidermal Growth Factor Receptor (EGFR) mutation in Asia [3]. Fortunately, in 2004, Lynch, et al. discovered that NSCLC patients harboring specific EGFR mutations experienced clinical responsiveness to the tyrosine kinase inhibitor (TKI) [4]. Later, several clinical trials demonstrated that advanced NSCLC patients with the EGFR mutation who had been receiving first- and second-generation EGFR-TKI treatment experienced longer progression-free survival (PFS) and fewer adverse effects than those patients receiving platinum-based chemotherapy [5,6,7]. Therefore, currently, the EGFR-TKIs are the standard first-line treatment for advanced NSCLC patients harboring sensitive EGFR mutation.

Osimertinib, a third-generation, irreversible, and selective EGFR-TKI, was designed to target the threonine 790 methionine (T790M) mutation, which is one of the resistance mechanisms associated with first- and second-generation EGFR-TKIs [8]. The phase 3 trial AURA 3 demonstrated that osimertinib significantly prolonged PFS compared to platinum-based chemotherapy in advanced EGFR-mutant NSCLC patients with the T790M mutation who had developed resistance to first- and second-generation EGFR-TKI treatments [9]. Furthermore, in a first-line setting, the FLAURA study demonstrated that EGFR-mutant advanced NSCLC patients who were treatment-naïve and taking an upfront osimertinib regimen experienced significantly better median PFS and OS than those who had been treated with the first-generation EGFR-TKIs gefitinib and erlotinib [10,11].

Although the FLAURA study confirmed that there were promising survival benefits of treatment with third-generation EGFR-TKI as a first-line treatment, several real-world studies have found that sequential osimertinib use could also achieve satisfying clinical outcomes for advanced EGFR-mutant NSCLC patients harboring acquired T790M mutation after experiencing progressive disease under first- and second-generation EGFR-TKIs. The GioTag study showed that patients with EGFR mutation-positive NSCLC who were receiving sequential afatinib and osimertinib experienced a median time on treatment of 27.7 months and a median OS of 37.6 months [12]. In the RESET study, sequential afatinib and osimertinib treatment in Asian patients with EGFR-mutant NSCLC who acquired the T790M mutation achieved an OS of 54.3 months [13]. In Taiwan, we also demonstrated that advanced EGFR-mutant NSCLC patients being treated with sequential osimertinib experienced an OS of more than 60 months if T790M mutation was noted after gaining resistance to first- and second-generation EGFR-TKIs [14]. Although several real-world-data studies have confirmed the clinical benefits of sequential osimertinib treatment, few have discussed the role of sequential osimertinib treatment in different treatment lines. Thus, we conducted the present study in order to investigate the possible difference in clinical outcomes between second-line osimertinib and ≥third-line treatment in advanced and recurrence EGFR-mutant NSCLC patients with acquired T790M mutation.

## 2. Material and Methods

### 2.1. Study Design and Patients

This study was a single-center, observational, retrospective analysis conducted at Taichung Veterans General Hospital (TCVGH) in Taiwan. It received approval from the Institutional Review Board (IRB) of TCVGH, Taiwan (IRB No. CE24125C).

We analyzed patients who had received sequential osimertinib treatment during the period from September 2014 to March 2023. In order to be enrolled for the study, patients had to fulfill the following seven inclusion criteria: (1) a diagnosis of NSCLC confirmed by histology or cytology; (2) recurrence or stage IV lung cancer, as defined by the 8th edition of the American Joint Committee on Cancer (AJCC) staging system; (3) the presence of an EGFR mutation, specifically exon 19 deletion or exon 21 L858R point mutation; (4) receiving first-line treatment with EGFR-TKIs, including gefitinib, erlotinib, or afatinib; (5) undergoing tissue or liquid rebiopsy after experiencing drug resistance to a first-line EGFR-TKI; (6) acquired T790M mutation detected by either a tissue or liquid rebiopsy; and (7) taking osimertinib as a sequential treatment. Patients were excluded if they met any of the following three criteria: (1) having EGFR mutations other than exon 19 deletion and L858R mutation; (2) receiving sequential osimertinib treatment involving combined chemotherapy or anti-angiogenic agents; and (3) being diagnosed with another active malignancy. Chest computed tomography was performed every three months to meet the requirements for National Health Insurance reimbursement. Treatment response to EGFR-TKIs was assessed using the Response Evaluation Criteria in Solid Tumors (Version 1.1).

Each patient’s demographic and clinical data, including age, gender, smoking status, Eastern Cooperative Oncology Group Performance Status (ECOG PS), clinical stage, EGFR mutation status at baseline, condition of brain metastasis at baseline, type of EGFR-TKI treatment, and treatment lines of osimertinib as well as their best response to EGFR-TKI, PFS, and OS for EGFR-TKI were all collected for analysis.

### 2.2. EGFR Mutation Test for Tumor Tissue and Liquid Biopsy

EGFR mutations in tumor tissue were analyzed using either the cobas^®^ EGFR Mutation Test v2 (Roche Molecular Systems, Pleasanton, CA, USA) or Matrix-Assisted Laser Desorption/Ionization Time-of-Flight Mass Spectrometry (MALDI-TOF MS). Plasma cell-free DNA extraction and T790M detection in liquid biopsies were performed following protocols from our previous studies, with some modifications [15]. A combination of peptide nucleic acid (PNA) and MALDI-TOF nucleotide mass spectrometry was used for experiments in an ISO15189-certified clinical center laboratory. PNA oligos, designed to selectively bind to the wild-type allele, were synthesized by PanaGene (Daejeon, Korea). All nucleotide MALDI-TOF MS assays were based on the MassARRAY System (Agena Bioscience, San Diego, CA, USA), following the manufacturer’s guidelines. Data collection and analysis were carried out using Typer 4 software (Agena Biosciences, USA). The tests were conducted at the National Center of Excellence for Clinical Trial and Research, National Taiwan University Hospital.

### 2.3. Statistical Analyses

We performed Fisher’s exact tests to evaluate the differences in patient characteristics between the second-line and ≥third-line treatment groups. Survival curves for PFS and OS were analyzed using the Kaplan–Meier method, with differences in survival times assessed by the log-rank test. All statistical analyses were conducted using SPSS 23.0 (SPSS Inc., Chicago, IL, USA). Two-tailed tests were used, and a *p*-value of <0.05 was considered statistically significant.

## 3. Results

### 3.1. Baseline Clinical Characteristics of Patients Undergoing Sequential Osimertinib Treatment

In total, 158 recurrent and stage IV NSCLC patients receiving osimertinib as sequential treatment were included for final analysis, with all of them harboring acquired T790M mutation after having experienced drug resistance to a first-line EGFR-TKI therapy that involved gefitinib, erlotinib, and afatinib (Figure S1). The Figure S2 presented the distribution of T790M mutation status detected through tissue and liquid biopsy. Patient characteristics and demographic data are shown in Table 1, with the median age being 62 years. There were 108 (68.4%) female patients, with only 21 (13.3%) patients being former or current smokers. The ECOG PS was 0–1 in 141 (89.2%) of the patients. Sixty (38.0%) patients were stage IVA, while seventy-seven (48.7%) were stage IVB. Twenty-one (13.3%) patients experienced disease recurrence after surgery. Eighty-six (54.4%) patients harbored EGFR exon 19 deletion at baseline, while seventy-two (45.6%) had exon 21 L858R point mutation. Fifty-nine (37.3%) patients experienced brain metastasis at baseline. Sixty-two (39.2%) patients received gefitinib, seventy-six (48.1%) received erlotinib, and twenty (12.7%) received afatinib as first-line EGFR-TKI treatments. Seventy-one (44.9%) patients underwent rebiopsy with tissue only, and fifty-five (34.8%) received both tissue and liquid biopsies.

### 3.2. The Comparison of Clinical Characteristics and Demographic Data Between Osimertinib as Second-Line and ≥Third-Line Treatment

There were 99 (62.7%) patients given osimertinib as second-line treatment (2nd-line group), and 59 (37.3%) were given osimertinib as ≥third-line treatment (≥3rd-line group). The patients’ clinical characteristics and demographic data, including age, gender, smoking status, ECOG PS, clinical stage, condition of brain metastasis at baseline, and the method of rebiopsy, did not reveal any differences between these two groups. Regarding baseline EGFR mutation, there were more patients harboring exon 21 L858R point mutation in the 2nd-line group (53.5%) than in the ≥3rd-line group (32.3%), whereas there were more patients diagnosed as having exon 19 deletion at baseline in the ≥3rd-line group (67.8%) than in the 2nd-line group (46.5%) (*p* = 0.013). Additionally, the type of first-line EGFR-TKI treatment was different between the 2nd-line and ≥3rd-line group (*p* = 0.023). In the 2nd-line group, 35 (35.4%) patients underwent gefitinib as first-line EGFR-TKI therapy, while 46 (46.5%) used erlotinib and 18 (18.1%) took afatinib. In the ≥3rd-line group, patients who received gefitinib numbered 27 (45.8%), those given erlotinib 30 (50.8%), and those treated with afatinib 2 (3.4%) for first-line treatment. The detailed analyses are summarized in Table 2.

### 3.3. Survival Outcomes in Patients Undergoing Sequential Osimertinib Treatment

**Figure 1:**
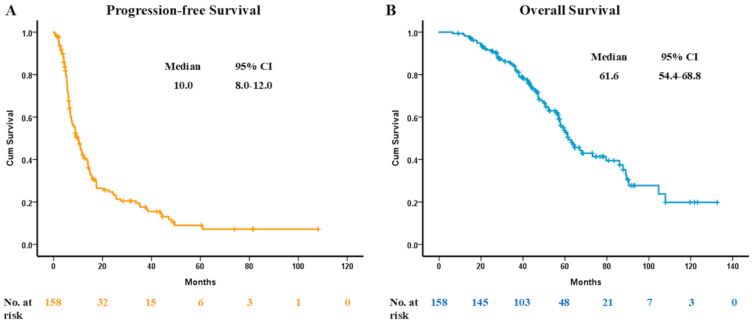
(**A**) The progression-free survival of osimertinib. (**B**) The overall survival from first-line EGFR-TKI. EGFR, epidermal growth factor receptor; TKI, tyrosine kinase inhibitor.

**Figure 2:**
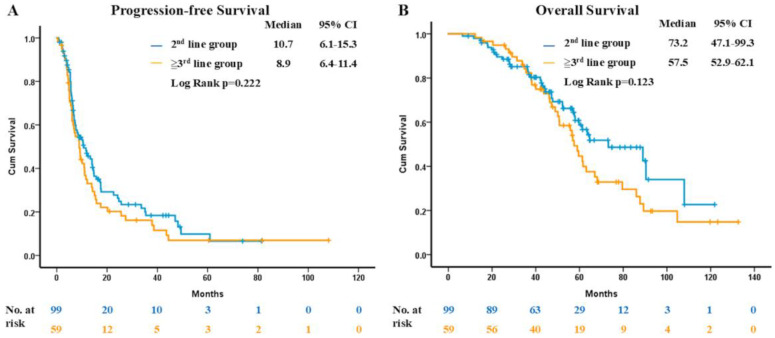
(**A**) The progression-free survival of osimertinib regarding different-line osimertinib treatment. (**B**) The overall survival from first-line EGFR-TKI regarding different-line osimertinib treatment. EGFR, epidermal growth factor receptor; TKI, tyrosine kinase inhibitor.

The end of follow-up date was 1 August 2023, with the median follow-up period being 48 months. The median PFS of osimertinib was 10.0 months (95% CI, 8.0 to 12.0) (Figure 1A). The median OS of osimertinib was 33.4 months (95% CI, 23.5 to 43.3), and the median OS from first-line EGFR-TKI was 61.6 months (95% CI, 54.4 to 68.8) (Figure 1B). Regarding the different lines of sequential treatment, the median PFS of osimertinib was 10.7 months (95% CI, 6.1 to 15.3) in the 2nd-line group and 8.9 months (95% CI, 6.4 to 11.4) in the ≥3rd-line group (Figure 2A). The median OS from first-line EGFR-TKI was 73.2 months (95% CI, 47.1 to 99.3) in the 2nd line group and 57.5 months (95% CI, 52.9 to 62.1) in the ≥3rd-line group (Figure 2B). Based on the log-rank test, the Kaplan–Meier curve did not show statistical difference in the PFS of osimertinib (*p* = 0.222) and OS from first line EGFR-TKI (*p* = 0.123) between the 2nd-line and ≥3rd-line groups. The survival curves of other patient characteristics are displayed in Figures S3–S10.

### 3.4. The Univariate and Multivariate Analysis of Survival Outcomes

The Cox proportional hazards model revealed that certain clinical factors, such as age, gender, smoking status, clinical stage, baseline brain metastasis status, and the type of first-line EGFR-TKI treatment, did not impact PFS outcomes. Only patients with exon 19 deletion at baseline experienced a trend of better PFS than those with exon 21 L858R point mutation [adjusted HR of 0.71 (95% CI, 0.48 to 1.06); *p* = 0.092-]. Additionally, the treatment lines of sequential osimertinib were not found to affect the PFS of osimertinib patients [adjusted HR of 0.81 (95% CI, 0.54 to 1.210); *p* = 0.303]. Through multivariate analysis, all clinical factors did not result in any different clinical efficacy of OS, with the exception of baseline EGFR mutation status. Patients harboring exon 19 deletion at baseline experienced longer OS than those who harbored exon 21 L858R point mutation [adjusted HR of 0.51 (95% CI, 0.30 to 0.85); *p* = 0.010]. Patients in the 2nd-line group did not have better OS than patients in the ≥3rd-line group [adjusted HR of 0.70 (95% CI, 0.43 to 1.17); *p* = 0.174]. The results of univariate and multivariate analysis for survival outcomes are shown in Table 3 and Table 4.

## 4. Discussion

Our research demonstrated the real-world efficacies of sequential osimertinib treatment in advanced and recurrence EGFR-mutant NSCLC patients harboring acquired T790M mutation after progressive disease to first-line EGFR-TKIs. Patients who received sequential osimertinib could successfully experience a median OS of more than 5 years. Additionally, patients could receive the equivalent clinical benefits of sequential osimertinib in both 2nd-line and ≥3rd-line treatment.

In the AURA3 study, the median PFS was significantly longer in the osimertinib group than in the chemotherapy group for patients with advanced T790M-positive NSCLC for whom disease had progressed during first-line EGFR-TKI therapy [10.1 months versus 4.4 months; HR of 0.30 (95% CI, 0.23 to 0.41); *p* < 0.001] [9]. The median OS was 26.8 months in the osimertinib group and 22.5 months in the chemotherapy group [16]. Additionally, the ASTRIS study, a global observational study enrolling more than 3000 patients for analysis, showed that the median PFS was 11.1 months for osimertinib patients in real-world practice [17]. In a subgroup analysis of the ASTRIS study performed in China, median PFS was 11.7 months (95% CI, 11.1 to 12.5), and median time-to-treatment discontinuation was 13.9 months (95% CI, 13.1 to 15.2) in that particular Chinese population [18]. The clinical efficacies of osimertinib from our research were comparable with that found in previous clinical trials and studies, with median PFS being 10.0 months (95% CI, 8.0 to 12.0) and median OS being 33.4 months (95% CI, 23.5 to 43.3).

Although the clinical benefits of sequential osimertinib have been confirmed through clinical trials and several real-world-data studies, there have been few studies investigating the relationship of the different-line therapies of osimertinib and their clinical outcomes. In the pooled analysis of the AURA extension and the AURA2 trial, 411 patients receiving sequential osimertinib treatment were included for analysis, with 129 patients in the 2nd-line group and 282 patients in the ≥3rd-line group [19]. The median PFS of osimertinib patients was 9.7 months (95% CI, 8.3 to 11.7) and 11.0 months (95% CI, 8.5 to 12.6) in the 2nd-line and the ≥3rd-line groups, respectively. In real-world practice, Chiang et al. presented patients who had received intercalated treatment prior to sequential osimertinib and had experienced a median PFS of 12.8 months (95% CI, 7.1 to 18.6), while the PFS of patients without intercalated therapy prior to sequential osimertinib treatment was 15.2 months (95% CI, 9.2 to 21.3) [20]. In the present study, we demonstrated that the median PFS of osimertinib patients was 10.7 months (95% CI, 6.1 to 15.3) in the 2nd-line group and 8.9 months (95% CI, 6.4 to 11.4) in the ≥3rd-line group. There was no statistical difference in PFS between the 2nd-line and ≥3rd-line groups, with an adjusted HR of 0.81 (95% CI, 0.54 to 1.21). Our findings are consistent with those of previous studies, with the present study involving a larger cohort than previous studies in a real-world setting. Thus, the evidence is stronger than in the other previously performed research in proving this phenomenon.

Currently, the revolution of first-line treatment for advanced EGFR-mutant NSCLC patients remains focused on third-generation EGFR-TKIs. According to the FLAURA study, advanced EGFR-mutant NSCLC patients receiving the third-generation EGFR-TKI osimertinib as first-line treatment could experience a significantly longer median PFS [18.9 months versus 10.2 months; HR of 0.46 (95% CI, 0.37 to 0.57); *p* < 0.001] as well as a longer median OS [38.6 months versus 31.8 months; HR of 0.80 (95% CI, 0.64 to 1.00); *p* = 0.046] compared to patients given first-generation EGFR-TKIs [10,11]. Another potent third-generation EGFR-TKI, lazertinib, was also proven to provide a median PFS that is significantly longer than gefitinib [20.6 months versus 9.7 months; HR of 0.45 (95% CI, 0.34 to 0.58); *p* < 0.001] in first-line treatment for EGFR-mutated advanced NSCLC patients [21]. Additionally, there have been certain clinical trials that have demonstrated the clinical efficacies of a combination regimen with third-generation EGFR-TKIs in the first-line setting. In the FLAURA2 trial, patients with EGFR-mutated advanced NSCLC who were given osimertinib plus a chemotherapy regimen experienced significantly longer PFS than those undergoing osimertinib monotherapy as first-line treatment [25.5 months versus 16.7 months; HR of 0.62 (95% CI, 0.49 to 0.79); *p* < 0.001] [22]. The MARIPOSA trial demonstrated that amivantamab plus lazertinib provided a meaningful clinically statistical improvement in PFS when compared to osimertinib [23.7 months versus 16.6 months; HR of 0.70 (95% CI, 0.58 to 0.85); *p* < 0.001] [23].

Although the third-generation EGFR-TKIs have been the favorable choice for treatment-naïve EGFR-mutated advanced NSCLC patients, the regimen of first- and second-generation EGFR-TKI upfront use followed by sequential third-generation EGFR-TKI treatment still remains an attractive option for both patients and clinicians. There have been several real-world studies showing promising OS data in patients who underwent sequential osimertinib therapy. In the RESET study, sequential afatinib and osimertinib treatment in Asian patients with EGFR-mutant NSCLC who acquired the T790M mutation experienced a median OS of 54.3 months [13]. The real-world data from Taiwan presented a median OS from first-line EGFR-TKI treatment of 57.0 to 61.3 months in advanced EGFR-positive NSCLC patients with acquired T790M mutation after progressive disease under first- and second-generation EGFR-TKIs with sequential osimertinib treatment [14,20]. Our present study also demonstrated that the median OS from first line EGFR-TKI was 61.6 months (95% CI, 54.4 to 68.8). Additionally, our data found that median OS was similar between the 2nd-line osimertinib use group and the ≥3rd-line osimertinib use group [73.2 months versus 57.5 months; adjusted HR of 0.70 (95% CI, 0.43 to 1.17); *p* = 0.174].

Despite the favorable OS outcomes resulting from sequential osimertinib therapy, the fact is that not all patients taking first- and second-generation EGFR-TKIs as first-line treatment will harbor acquired T790M mutation after drug resistance has developed. A previous study showed that the secondary EGFR mutation, the T790M mutation, accounted for around 60% of the resistance mechanisms to first- and second-generation EGFR-TKIs [24]. To increase the yield rate of acquired T790M mutation, Ninomaru et al. emphasized the importance of repeated rebiopsy [25]. Within a 50-patient cohort, the T790M mutation was detected within 18 patients upon first-time rebiopsy, with 22 patients noted to have an acquired T790M mutation after undergoing repeated rebiopsy. Ultimately, the overall yield rate of T790M mutation was 80%. Chiang et al. also noted a 36.8% positive rate of T790M mutation in patients undergoing second-time rebiopsy [20]. It is possible that the heterogenicity of tumor cells may result in the condition. Thus, we should encourage patients to undergo a rebiopsy after progressive disease under first- and second-generation EGFR-TKIs if the possible approach site for rebiopsy is safe. Alternatively, there have been studies that used chest computed tomography and brain magnetic resonance imaging-based radiomics to predict T790M mutation after first- and second-generation EGFR-TKI treatment [26,27]. If we are able to apply radiomic based artificial intelligence to predict the chance of T790M mutation occurring prior to first-line EGFR-TKI treatment, then clinicians can more easily select any patient suitable to adopt a sequential osimertinib regimen. However, further research is still necessary in order to confirm our theory.

To the best of our knowledge, the present study is the first to investigate the impact of the different lines of sequential osimertinib on clinical efficacy as the primary end point in advanced and recurrent EGFR-mutant NSCLC patients with acquired T790M mutation. However, this study has still some limitations. First, this study was a single-center, retrospective analysis, which may have introduced more bias compared to prospectively designed studies. Second, only Taiwanese individuals were included in the analysis, so the findings may not be applicable to other ethnic populations. Third, there was a little difference in patient characteristics and demographic data between the 2nd-line group and ≥3rd-line group, so it became necessary to use the multivariate analysis to overcome the selection bias.

## 5. Conclusions

Our findings shed light on the clinical efficacy of osimertinib as a form of sequential therapy after progressive disease under first- and second-generation EGFR-TKI in advanced and recurrence EGFR-mutant NSCLC patients with acquired T790M mutation as well as the impact of the different lines of sequential osimertinb used. In conclusion, our research demonstrated that patients who underwent sequential osimertinib treatment experienced satisfactory PFS and OS. Additionally, we found that not only 2nd-line osimertinib treatment but also ≥3rd-line osimertinib treatment provided sufficient efficacies in advanced and recurrence EGFR-mutant NSCLC patients harboring T790M mutation after experiencing drug resistance to first- and second-generation EGFR-TKIs.

## Figures and Tables

**Table 1:** Patients’ characteristics and demographic data.

Characteristics	*n* = 158
Age (years), median (range)	62 (32–92)
Gender, *n* (%)	
Male	50 (31.6)
Female	108 (68.4)
Smoking status, *n* (%)	
Non-smokers	137 (86.7)
Former or current smokers	21 (13.3)
ECOG PS, *n* (%)	
0–1	141 (89.2)
2–4	17 (10.8)
Stage	
IVA	60 (38.0)
IVB	77 (48.7)
Recurrence	21 (13.3)
EGFR subtype, *n* (%)	
Exon 19 deletion	86 (54.4)
Exon 21 L858R point mutation	72 (45.6)
Brain metastasis at baseline, *n* (%)	
With brain metastasis	59 (37.3)
Without brain metastasis	99 (62.7)
First-line EGFR-TKI, *n* (%)	
Gefitinib	62 (39.2)
Erlotinib	76 (48.1)
Afatinib	20 (12.7)
The method of rebiopsy	
Tissue biopsy	71 (44.9)
Liquid biopsy	32 (20.3)
Both	55 (34.8)
Treatment line of osimertinib, *n* (%)	
Second-line treatment	99 (62.7)
≥Third-line treatment	59 (37.3)

ECOG PS, Eastern Cooperative Oncology Group performance status; EGFR, Epidermal Growth Factor Receptor; TKI, Tyrosine Kinase Inhibitor.

**Table 2:** Comparison of the clinical characteristics between osimertinib as 2nd-line treatment and ≥3rd-line treatment.

	Osimertinib		
Characteristics	2nd-Line, *n* (%)	≥3rd-Line, *n* (%)	*p*-Value *
Age			0.069
≥65	49 (49.5)	20 (33.9)	
<65	50 (50.5)	39 (66.1)	
Gender, *n* (%)			0.861
Male	32 (32.3)	18 (30.5)	
Female	67 (67.7)	41 (69.5)	
Smoking status, *n* (%)			1.000
Non-smokers	86 (86.9)	51 (86.4)	
Former or current smokers	13 (13.1)	8 (13.6)	
ECOG PS, *n* (%)			0.291
0–1	86 (86.9)	55 (93.2)	
2–3	13 (13.1)	4 (6.8)	
Stage, *n* (%)			0.177
Post-operation recurrence	17 (17.1)	4 (6.8)	
Stage 4A	36 (36.4)	24 (40.7)	
Stage 4B	46 (46.5)	31 (52.5)	
Baseline EGFR mutation status, *n* (%)			0.013
Exon 19 deletions	46 (46.5)	40 (67.8)	
Exon 21 L858R	53 (53.5)	19 (32.2)	
Brain metastasis at baseline, *n* (%)			0.126
Yes	32 (32.3)	27 (45.8)	
No	67 (67.7)	32 (54.2)	
First-line EGFR-TKI, *n* (%)			0.023
Gefitinib	35 (35.4)	27 (45.8)	
Erlotinib	46 (46.5)	30 (50.8)	
Afatinib	18 (18.1)	2 (3.4)	
The method of rebiopsy			0.384
Tissue biopsy	41 (41.4)	30 (50.8)	
Liquid biopsy	23 (23.2)	9 (15.3)	
Both	35 (35.4)	20 (33.9)	

ECOG PS, Eastern Cooperative Oncology Group performance status; EGFR, epidermal growth factor receptor; TKI, tyrosine kinase inhibitor; * by Fisher’s exact test.

**Table 3:** Univariate and multivariate analysis of progression-free survival.

Characteristics	HR (95% CI) *	*p*-Value	Adjusted HR (95% CI) *	*p*-Value
Age (years) ≥65 <65	Reference 1.37 (0.95–1.98)	0.093	1.49 (0.97–2.28)	0.070
Gender Female Male	Reference 1.06 (0.72–1.56)	0.780	1.18 (0.71–1.95)	0.528
Smoking status C/FS NS	Reference 0.78 (0.46–1.32)	0.358	0.80 (0.41–1.53)	0.496
ECOG PS 2–4 0–1	Reference 0.62 (0.37–1.05)	0.074	0.66 (0.37–1.16)	0.145
Stage Recurrence Stage IVA Stage IVB	Reference 1.50 (0.83–2.73) 1.82 (1.02–3.27)	0.182 0.044	1.47 (0.78–2.77) 1.67 (0.87–3.20)	0.239 0.123
EGFR subtype Exon 21 L858R point mutation Exon 19 deletion	Reference 0.90 (0.63–1.29)	0.559	0.71 (0.48–1.06)	0.092
Brain metastasis at baseline With brain metastasis Without brain metastasis	Reference 0.80 (0.56–1.15)	0.224	0.88 (0.57–1.38)	0.585
First-line EGFR-TKI Afatinib Gefitinib Erlotinib	Reference 1.13 (0.60–2.13) 1.22 (0.66–2.28)	0.703 0.523	1.27 (0.63–2.57) 1.04 (0.54–2.03)	0.505 0.899
Treatment line of osimertinib ≥Third-line treatment Second-line treatment	Reference 0.80 (0.56–1.15)	0.225	0.81 (0.54–1.21)	0.303

HR, hazard ratio; CI, confidence interval; C/FS, current/former smokers; NS, non-smoker; ECOG PS, Eastern Cooperative Oncology Group performance status; EGFR, Epidermal Growth Factor Receptor; TKI, Tyrosine Kinase Inhibitor; * by Cox proportional hazard model.

**Table 4:** Univariate and multivariate analysis of overall survival.

Characteristics	HR (95% CI) *	*p*-Value	Adjusted HR (95% CI) *	*p*-Value
Age (years) ≥65 <65	Reference 1.41 (0.89–2.25)	0.148	1.52 (0.89–2.60)	0.127
Gender Female Male	Reference 1.38 (0.85–2.23)	0.188	1.25 (0.65–2.38)	0.504
Smoking status C/FS NS	Reference 0.51 (0.28–0.94)	0.029	0.55 (0.25–1.23)	0.144
ECOG PS 2–4 0–1	Reference 0.73 (0.34–1.60)	0.431	1.05 (0.45–2.41)	0.918
Stage Recurrence Stage IVA Stage IVB	Reference 0.96 (0.46-2.00) 1.98 (0.99–3.98)	0.913 0.055	0.98 (0.45–2.13) 1.82 (0.84–3.92)	0.954 0.128
EGFR subtype Exon 21 L858R point mutation Exon 19 deletion	Reference 0.73 (0.47–1.14)	0.167	0.51 (0.30–0.85)	0.010
Brain metastasis at baseline With brain metastasis Without brain metastasis	Reference 0.63 (0.40–1.00)	0.049	0.85 (0.50–1.43)	0.538
First-line EGFR-TKI Afatinib Gefitinib Erlotinib	Reference 0.94 (0.39–2.27) 1.42 (0.60–3.36)	0.886 0.424	1.00 (0.39–2.58) 1.16 (0.48–2.82)	0.505 0.743
Treatment line of osimertinib ≥Third-line treatment Second-line treatment	Reference 0.70 (0.45–1.10)	0.125	0.70 (0.43–1.17)	0.174

HR, hazard ratio; CI, confidence interval; C/FS, current/former smokers; NS, non-smoker; ECOG PS, Eastern Cooperative Oncology Group performance status; EGFR, Epidermal Growth Factor Receptor; TKI, Tyrosine Kinase Inhibitor; * by Cox proportional hazard model.

## Data Availability

The original contributions presented in this study are included in the article/Supplementary Material. Further inquiries can be directed to the corresponding author.

## Supplementary Materials

The following supporting information can be downloaded at: https://www.mdpi.com/article/10.3390/cancers16244174/s1, Figure S1. Patient collection flowchart; Figure S2. The distribution of T790M mutations status detected through tissue and liquid biopsy; Figure S3. The survival outcomes of osimertinib based on age; Figure S4. The survival outcomes of osimertinib based on gender; Figure S5. The survival outcomes of osimertinib based on smoking status; Figure S6. The survival outcomes of osimertinib based on ECOG PS; Figure S7. The survival outcomes of osimertinib based on the stage; Figure S8. The survival outcomes of osimertinib based on *EGFR* mutation status; Figure S9. The survival outcomes of osimertinib based on brain metastasis status; Figure S10. The survival outcomes of osimertinib based on the first-line EGFR-TKI.

## References

[1] Kris M.G., Johnson B.E., Berry L.D., Kwiatkowski D.J., Iafrate A.J., Wistuba I.I., Varella-Garcia M., Franklin W.A., Aronson S.L., Su P.F. (2014). Using multiplexed assays of oncogenic drivers in lung cancers to select targeted drugs. JAMA.

[2] Jordan E.J., Kim H.R., Arcila M.E., Barron D., Chakravarty D., Gao J., Chang M.T., Ni A., Kundra R., Jonsson P. (2017). Prospective Comprehensive Molecular Characterization of Lung Adenocarcinomas for Efficient Patient Matching to Approved and Emerging Therapies. Cancer Discov..

[3] Shi Y., Au J.S., Thongprasert S., Srinivasan S., Tsai C.M., Khoa M.T., Heeroma K., Itoh Y., Cornelio G., Yang P.C. (2014). A Prospective, Molecular Epidemiology Study of EGFR Mutations in Asian Patients with Advanced Non-Small-Cell Lung Cancer of Adenocarcinoma Histology (PIONEER). J. Thorac. Oncol..

[4] Lynch T.J., Bell D.W., Sordella R., Gurubhagavatula S., Okimoto R.A., Brannigan B.W., Harris P.L., Haserlat S.M., Supko J.G., Haluska F.G. (2004). Activating Mutations in the Epidermal Growth Factor Receptor Underlying Responsiveness of Non-Small-Cell Lung Cancer to Gefitinib. N. Engl. J. Med..

[5] Mok T.S., Wu Y.L., Thongprasert S., Yang C.H., Chu D.T., Saijo N., Sunpaweravong P., Han B., Margono B., Ichinose Y. (2009). Gefitinib or Carboplatin-Paclitaxel in Pulmonary Adenocarcinoma. N. Engl. J. Med..

[6] Rosell R., Carcereny E., Gervais R., Vergnenegre A., Massuti B., Felip E., Palmero R., Garcia-Gomez R., Pallares C., Sanchez J.M. (2012). Erlotinib versus Standard Chemotherapy as First-Line Treatment for European Patients with Advanced EGFR Mutation-Positive Non-Small-Cell Lung Cancer (EURTAC): A Multicentre, Open-Label, Randomised Phase 3 Trial. Lancet Oncol..

[7] Sequist L.V., Yang J.C., Yamamoto N., O’Byrne K., Hirsh V., Mok T., Geater S.L., Orlov S., Tsai C.M., Boyer M. (2013). Phase III Study of Afatinib or Cisplatin Plus Pemetrexed in Patients with Metastatic Lung Adenocarcinoma with EGFR Mutations. J. Clin. Oncol..

[8] Cross D.A., Ashton S.E., Ghiorghiu S., Eberlein C., Nebhan C.A., Spitzler P.J., Orme J.P., Finlay M.R., Ward R.A., Mellor M.J. (2014). AZD9291, an Irreversible EGFR TKI, Overcomes T790M-Mediated Resistance to EGFR Inhibitors in Lung Cancer. Cancer Discov..

[9] Mok T.S., Wu Y.-L., Ahn M.-J., Garassino M.C., Kim H.R., Ramalingam S.S., Shepherd F.A., He Y., Akamatsu H., Theelen W.S. (2017). Osimertinib or Platinum-Pemetrexed in EGFR T790M-Positive Lung Cancer. N. Engl. J. Med..

[10] Ramalingam S.S., Vansteenkiste J., Planchard D., Cho B.C., Gray J.E., Ohe Y., Zhou C., Reungwetwattana T., Cheng Y., Chewaskulyong B. (2020). Overall Survival with Osimertinib in Untreated, EGFR-Mutated Advanced NSCLC. N. Engl. J. Med..

[11] Soria J.C., Ohe Y., Vansteenkiste J., Reungwetwattana T., Chewaskulyong B., Lee K.H., Dechaphunkul A., Imamura F., Nogami N., Kurata T. (2018). Osimertinib in Untreated EGFR-Mutated Advanced Non-Small-Cell Lung Cancer. N. Engl. J. Med..

[12] Hochmair M.J., Morabito A., Hao D., Yang C.T., Soo R.A., Yang J.C., Gucalp R., Halmos B., Märten A., Cufer T. (2020). Sequential Afatinib and Osimertinib in Patients with EGFR Mutation-Positive Non-Small-Cell Lung Cancer: Final Analysis of the GioTag Study. Future Oncol..

[13] Kim T., Jang T.W., Choi C.M., Kim M.H., Lee S.Y., Chang Y.S., Lee K.Y., Kim S.J., Yang S.H., Ryu J.S. (2023). Final Report on Real-World Effectiveness of Sequential Afatinib and Osimertinib in EGFR-Positive Advanced Non-Small Cell Lung Cancer: Updated Analysis of the RESET Study. Cancer Res. Treat..

[14] Huang Y.H., Tseng J.S., Hsu K.H., Chen K.C., Su K.Y., Yu S.L., Chen J.J.W., Yang T.Y., Chang G.C. (2021). The Impact of Different First-Line EGFR-TKIs on the Clinical Outcome of Sequential Osimertinib Treatment in Advanced NSCLC with Secondary T790M. Sci. Rep..

[15] Su K.Y., Tseng J.S., Liao K.M., Yang T.Y., Chen K.C., Hsu K.H., Yang P.C., Yu S.L., Chang G.C. (2018). Mutational Monitoring of EGFR T790M in cfDNA for Clinical Outcome Prediction in EGFR-Mutant Lung Adenocarcinoma. PLoS ONE.

[16] Papadimitrakopoulou V.A., Mok T.S., Han J.Y., Ahn M.J., Delmonte A., Ramalingam S.S., Kim S.W., Shepherd F.A., Laskin J., He Y. (2020). Osimertinib versus Platinum-Pemetrexed for Patients with EGFR T790M Advanced NSCLC and Progression on a Prior EGFR-Tyrosine Kinase Inhibitor: AURA3 Overall Survival Analysis. Ann. Oncol..

[17] Marinis F., Wu Y.L., de Castro G., Chang G.C., Chen Y.M., Cho B.C., Freitas H.C., Jiang L., Kim S.W., Martin C. (2019). ASTRIS: A Global Real-World Study of Osimertinib in >3000 Patients with EGFR T790M Positive Non-Small-Cell Lung Cancer. Future Oncol..

[18] Zhou Q., Zhang H.L., Jiang L.Y., Shi Y.K., Chen Y., Yu J.M., Zhou C.C., He Y., Hu Y.P., Liang Z.A. (2023). Real-World Evidence of Osimertinib in Chinese Patients with EGFR T790M-Positive Non-Small Cell Lung Cancer: A Subgroup Analysis from ASTRIS Study. J. Cancer Res. Clin. Oncol..

[19] Ahn M.J., Tsai C.M., Shepherd F.A., Bazhenova L., Sequist L.V., Hida T., Yang J.C.H., Ramalingam S.S., Mitsudomi T., Jänne P.A. (2019). Osimertinib in Patients with T790M Mutation-Positive, Advanced Non-Small Cell Lung Cancer: Long-Term Follow-Up from a Pooled Analysis of 2 Phase 2 Studies. Cancer.

[20] C Chiang C.L., Huang H.C., Shen C.I., Luo Y.H., Chen Y.M., Chiu C.H. (2020). Post-Progression Survival in Secondary EGFR T790M-Mutated Non-Small-Cell Lung Cancer Patients with and without Osimertinib after Failure of a Previous EGFR TKI. Target Oncol..

[21] Cho B.C., Ahn M.J., Kang J.H., Soo R.A., Reungwetwattana T., Yang J.C., Cicin I., Kim D.W., Wu Y.L., Lu S. (2023). Lazertinib versus Gefitinib as First-Line Treatment in Patients with EGFR-Mutated Advanced Non-Small-Cell Lung Cancer: Results from LASER301. J. Clin. Oncol..

[22] Planchard D., Jänne P.A., Cheng Y., Yang J.C., Yanagitani N., Kim S.W., Sugawara S., Yu Y., Fan Y., Geater S.L. (2023). Osimertinib with or without Chemotherapy in EGFR-Mutated Advanced NSCLC. N. Engl. J. Med..

[23] Cho B.C., Lu S., Felip E., Spira A.I., Girard N., Lee J.S., Lee S.H., Ostapenko Y., Danchaivijitr P., Liu B. (2024). Amivantamab Plus Lazertinib in Previously Untreated EGFR-Mutated Advanced NSCLC. N. Engl. J. Med..

[24] Yu H.A., Arcila M.E., Rekhtman N., Sima C.S., Zakowski M.F., Pao W., Kris M.G., Miller V.A., Ladanyi M., Riely G.J. (2013). Analysis of Tumor Specimens at the Time of Acquired Resistance to EGFR-TKI Therapy in 155 Patients with EGFR-Mutant Lung Cancers. Clin. Cancer Res..

[25] Ninomaru T., Hata A., Kokan C., Okada H., Tomimatsu H., Ishida J. (2021). Higher Osimertinib Introduction Rate Achieved by Multiple Repeated Rebiopsy after Acquired Resistance to First/Second Generation EGFR-TKIs. Thorac Cancer.

[26] Li Y., Lv X., Wang Y., Xu Z., Lv Y., Hou D. (2023). CT-Based Nomogram for Early Identification of T790M Resistance in Metastatic Non-Small Cell Lung Cancer Before First-Line Epidermal Growth Factor Receptor-Tyrosine Kinase Inhibitors Therapy. Eur. Radiol. Exp..

[27] Li Y., Lv X., Wang B., Xu Z., Wang Y., Sun M., Hou D. (2023). Predicting EGFR T790M Mutation in Brain Metastases Using Multisequence MRI-Based Radiomics Signature. Acad. Radiol..

