# Supplementary material for: Osimertinib as Second- and ≥Third-Line Treatment in Advanced and Recurrence EGFR-Mutant NSCLC Patients Harboring Acquired T790M Mutation
Source: Cancers (Basel). 2024 Dec 14;16(24):4174. doi: 10.3390/cancers16244174 (PMC11674345; doi:10.3390/cancers16244174)
Supplement: Supplementary file 1 [file cancers-16-04174-s001.zip › cancers-3331098-supplementary.pdf]

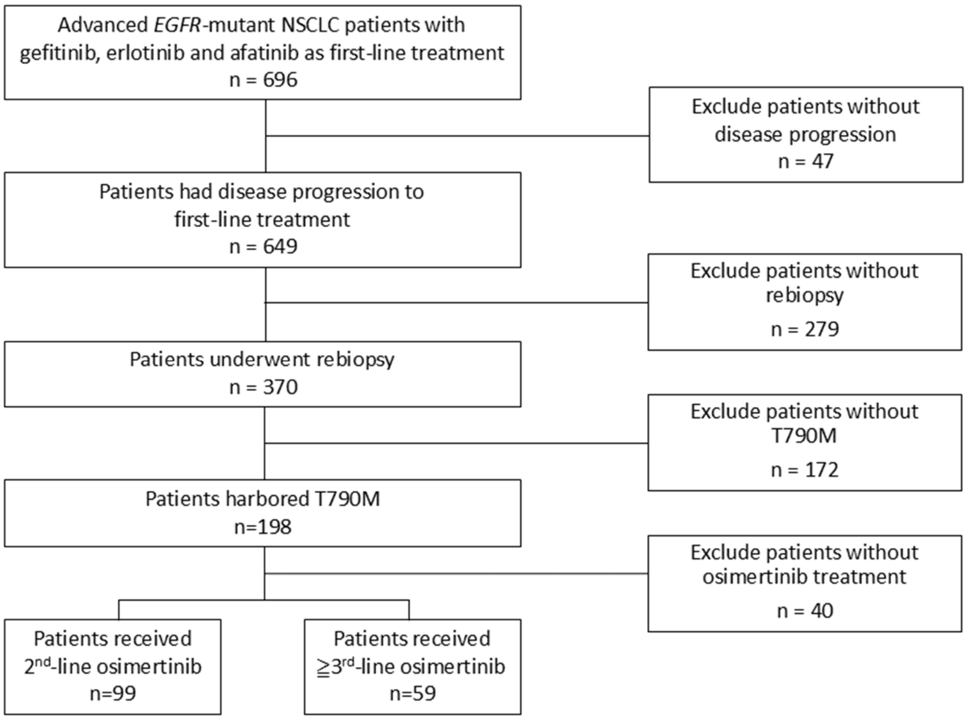

Figure S1. Patient collection flowchart. EGFR, epidermal growth factor receptor; NSCLC, non-small cell lung cancer.

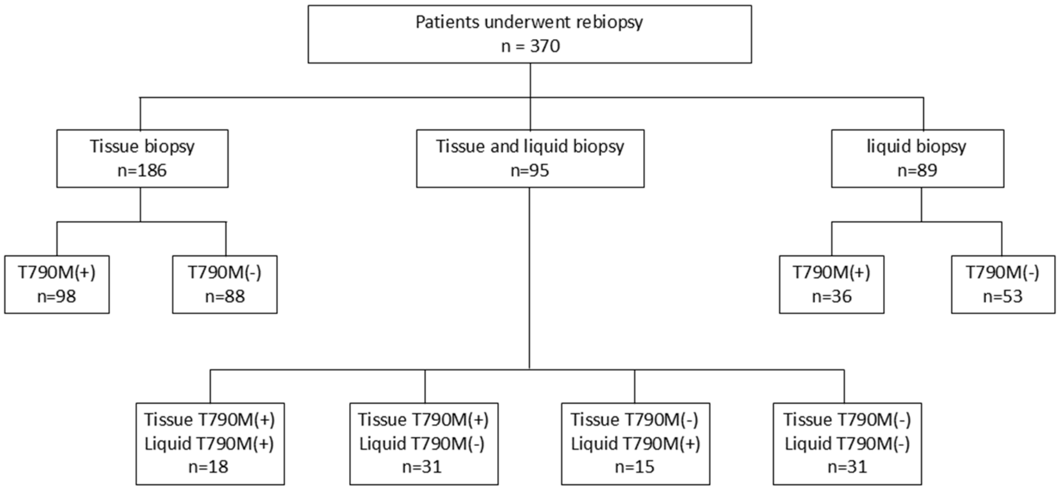

Figure S2. The distribution of T790M mutations status detected through tissue and liquid biopsy.

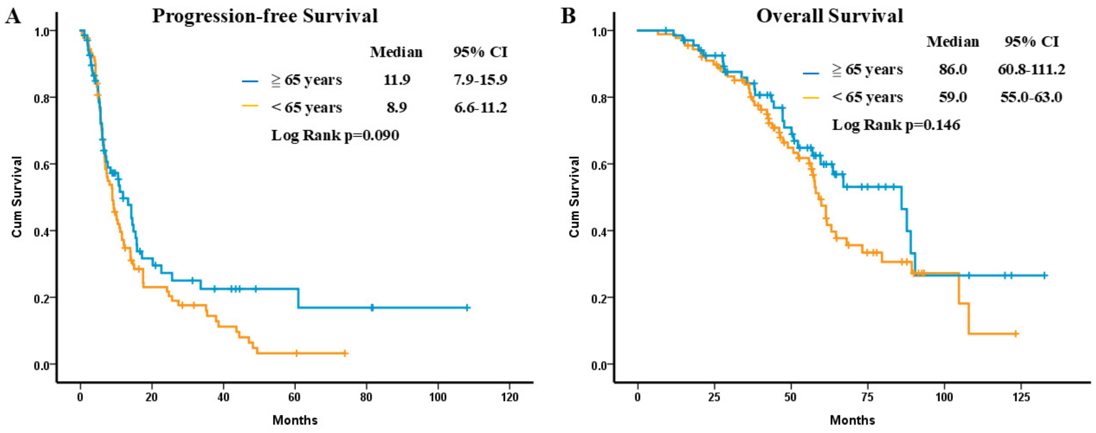

Figure S3. The survival outcomes of osimertinib based on age. A. Progression-free survival. B. Overall survival.

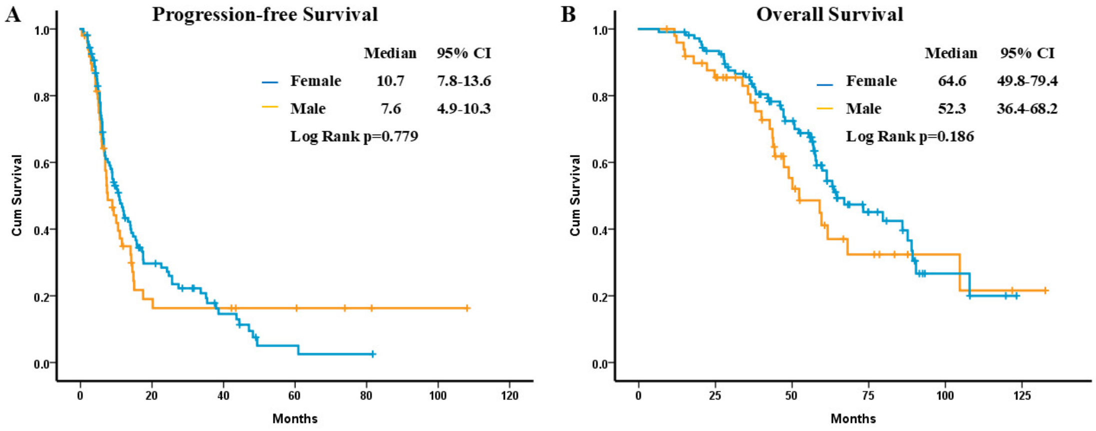

Figure S4. The survival outcomes of osimertinib based on gender. A. Progression-free survival. B. Overall survival.

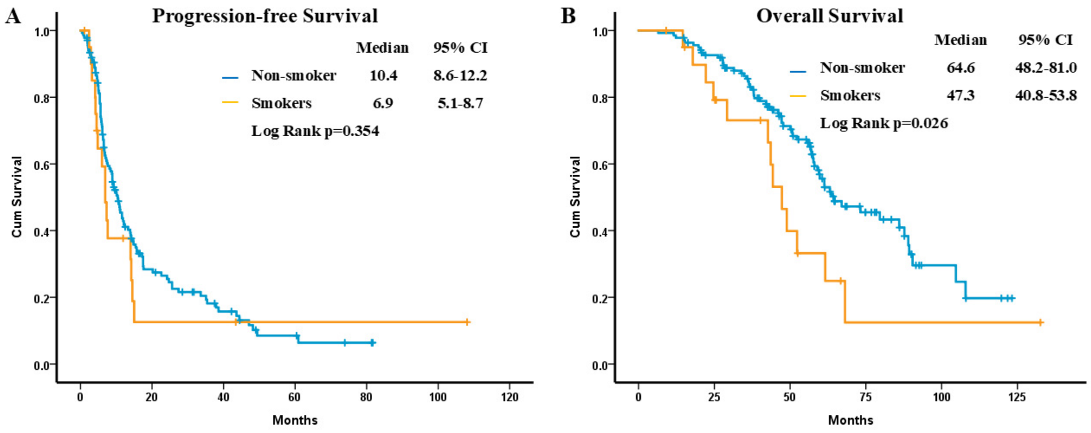

Figure S5. The survival outcomes of osimertinib based on smoking status. A. Progression-free survival. B. Overall survival.

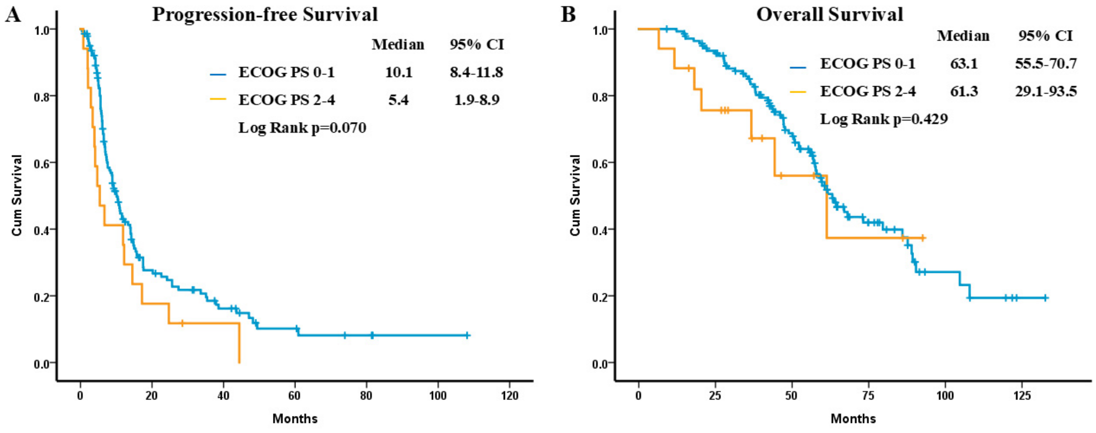

Figure S6. The survival outcomes of osimertinib based on ECOG PS. A. Progression-free survival. B. Overall survival. ECOG PS, Eastern Cooperative Oncology Group performance status.

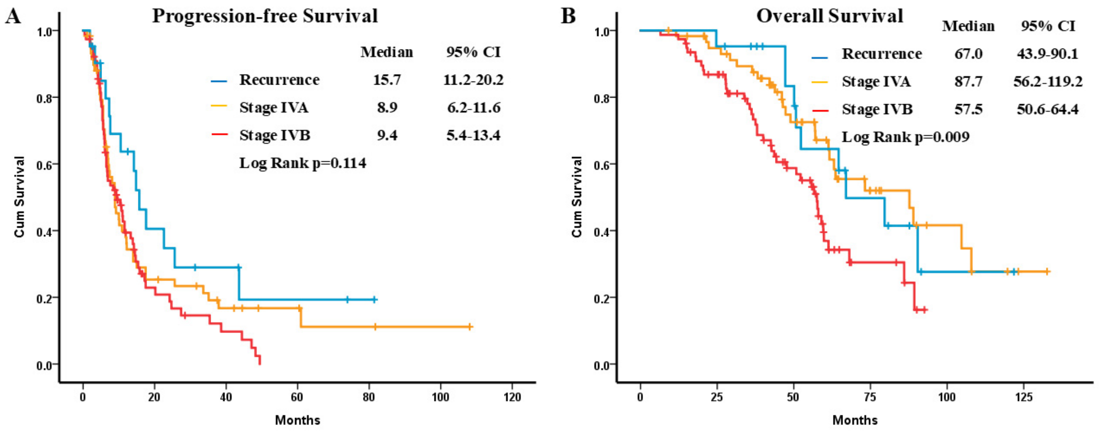

Figure S7. The survival outcomes of osimertinib based on the stage. A. Progression-free survival. B. Overall survival.

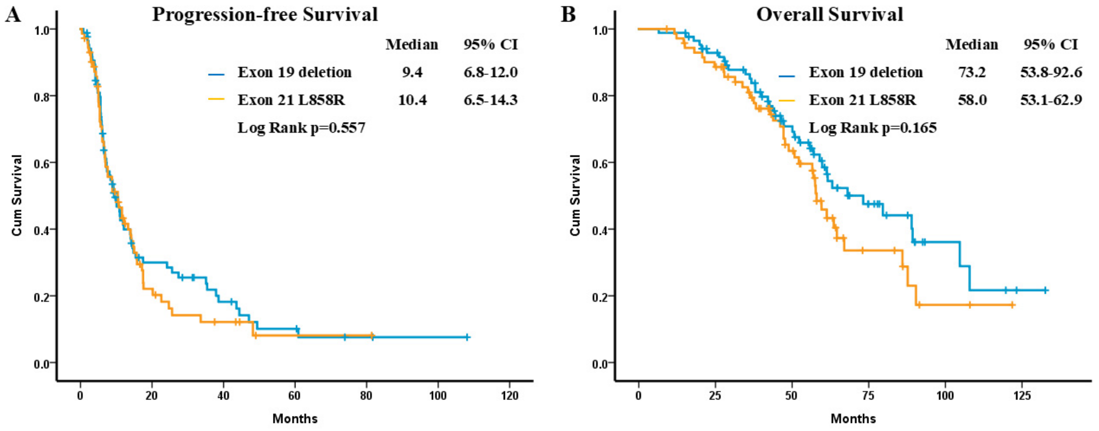

Figure S8. The survival outcomes of osimertinib based on *EGFR* mutation status. A. Progression-free survival. B. Overall survival. EGFR, epidermal growth factor receptor.

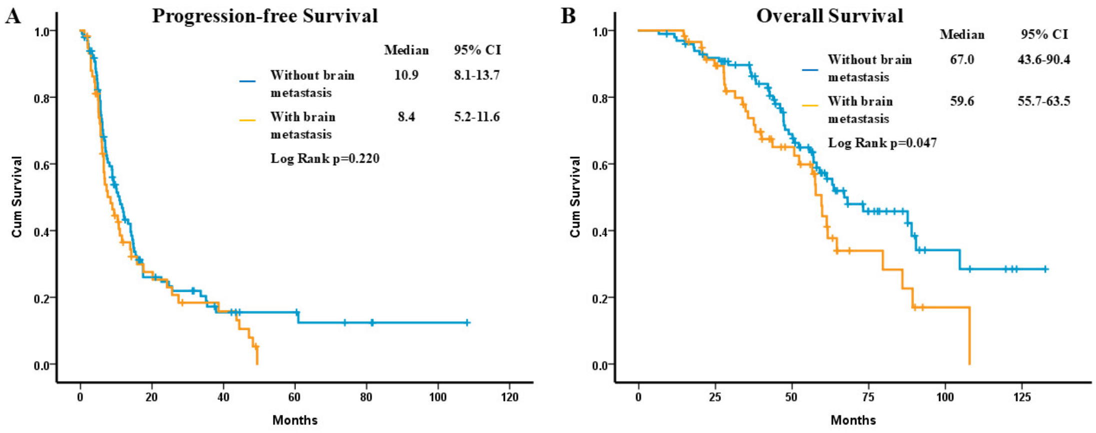

Figure S9. The survival outcomes of osimertinib based on brain metastasis status. A. Progression-free survival. B. Overall survival.

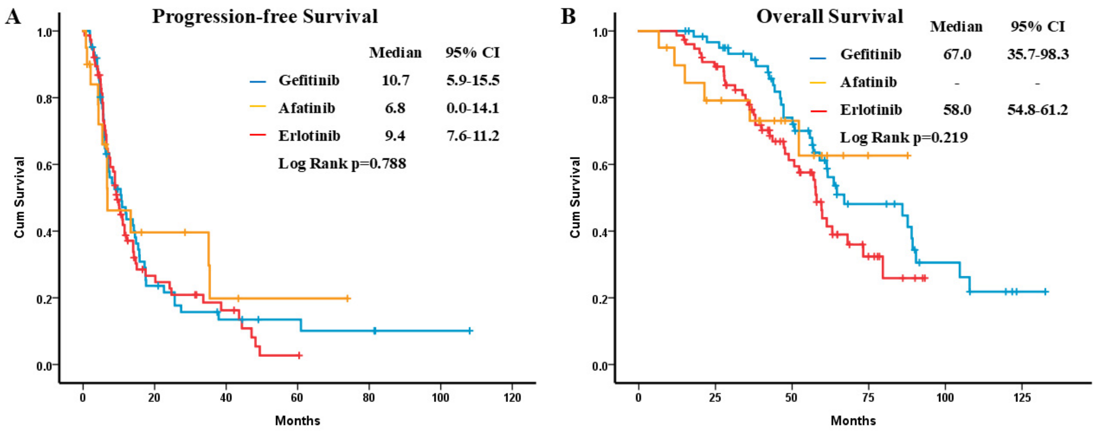

Figure S10. The survival outcomes of osimertinib based on the first-line EGFR-TKI. A. Progression-free survival. B. Overall survival. EGFR, epidermal growth factor receptor; TKI, tyrosine kinase inhibitor.
